# *Zyzyura*, a new genus of Eupatorieae (Asteraceae) from Belize

**DOI:** 10.3897/phytokeys.20.4033

**Published:** 2013-01-15

**Authors:** Harold Robinson, John F. Pruski

**Affiliations:** 1 Department of Botany, MRC 166, NMNH, P.O. Box 37012, Smithsonian, Washington DC. 20013,7012; 2 Missouri Botanical Garden, P.O. Box 299, St. Louis, Missouri, 63166

**Keywords:** *Zyzyura*, New Genus, *Fleischmannia*, Eupatorieae, Belize

## Abstract

A new Genus, *Zyzyura* is named to accommodate *Fleischmannia mayana* Pruski that has an eximbricate involucre, a high-conical receptacle, a corolla with a slender base closely investing the style and with a broadly campanulate limb, enlarged elongate cells in the carpopodium, short and broad distally protruding cells in the corolla lobes, and broad rounded anther appendages.

## Introduction

**Figure 1.**
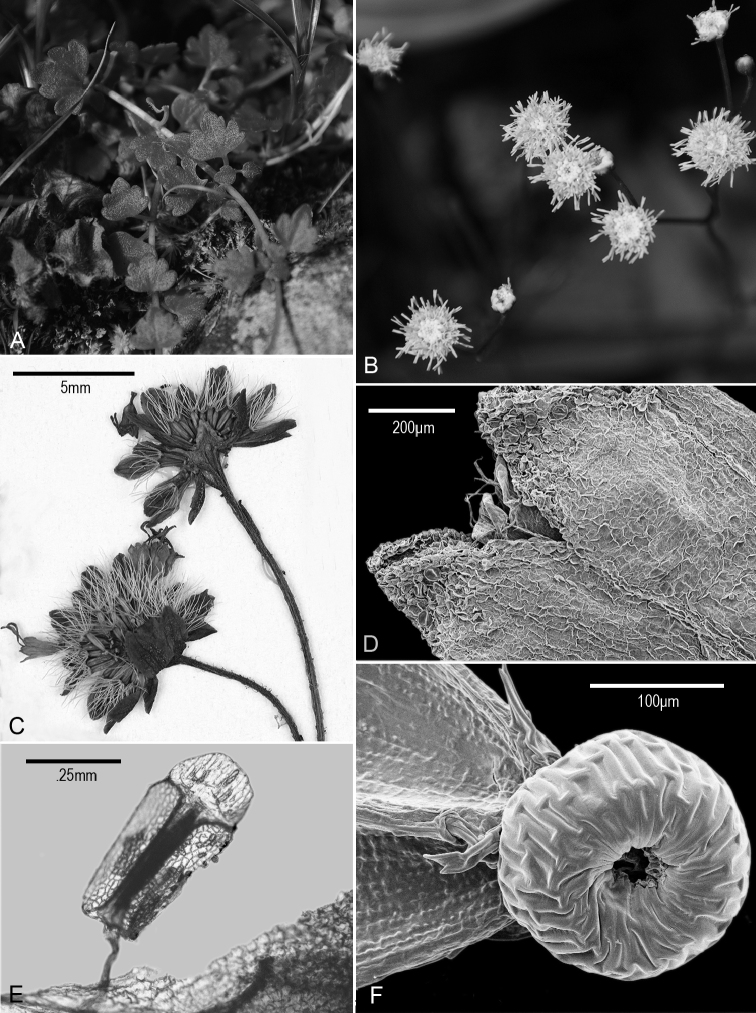
*Zyzyura mayana* (Pruski) H. Rob. & Pruski, **A** Prostrate portion of living plants showing lobed leaves **B** Capitula on erect flowering branches of living plant showing denser floral material near centers of capitula **C** Capitula of pressed specimen, lower capitulum showing eximbricate involucral bracts, upper head split, showing conical fistulose receptacle, upper capitulum also showing loose corolla **D** SEM of corolla lobes showing surfaces with protruding cells **E** Anther showing short theca and broad apical appendage **F** SEM micrograph of carpopodium showing elongate cells. (All from *Brewer & Pau 3349*)

**Figure 2.**
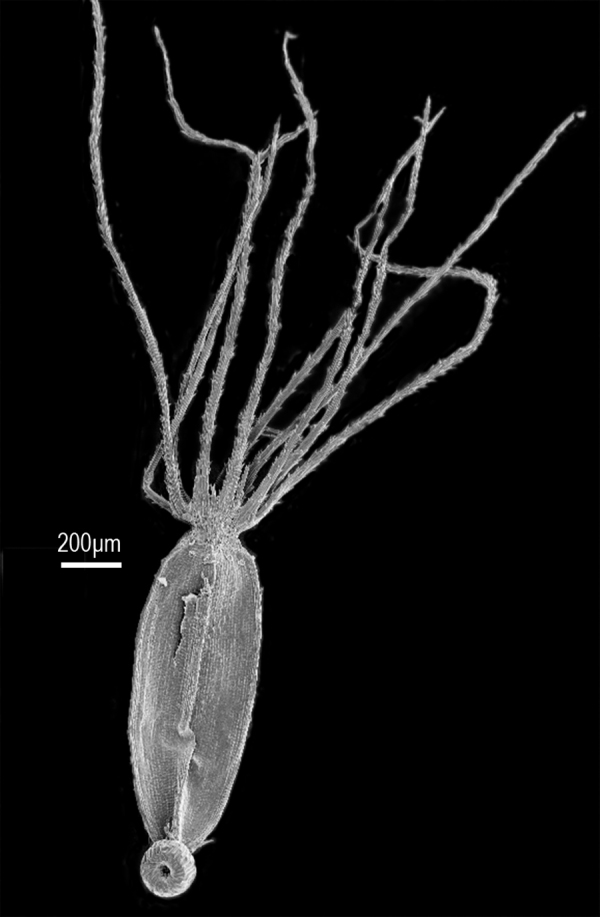
*Zyzyura mayana*, cypsela showing pappus of c. 10 non-contiguous bristles. (From *Brewer & Pau 3349*)

The recent description of *Fleischmannia mayana* Pruski (Pruski and Clase 2012) (Figs 1, 2) was based on material of a procumbent epilithic herb from the western slope of Victoria Peak in the Cockscomb Range of the Maya Mountains in Belize. The type specimen includes four segments of prostrate stems with ascending flowering branches and accompanying photos of the plants in the field. Details of the plants that are visible are essentially consistent throughout. In the initial study by Pruski and Clase (2012), using a manuscript key to the Eupatorieae of Mesoamerica (Robinson in press), the species was identified as a member of the genus *Fleischmannia*, and it was described in that genus. Now the type specimen has been subjected to a more intense study by the present authors, and a number of characteristics have been seen that preclude a position in *Fleischmannia* (King and Robinson 1987, Robinson 2001).

The concept of *Fleischmannia* Sch.Bip. has been well established since the redefinition of the limits of the genus (King and Robinson 1966, 1970) and in subsequent studies (King and Robinson 1972, 1974, 1975, 1978, 1991, Robinson 2001, 2011). The concept is now based on more than 90 species showing great uniformity in floral characteristics.

Four characteristics of *Fleischmannia mayana* are inconsistent with the characters that define *Fleischmannia* or even its only recognized close relative, *Sartorina* R.M.King & H.Rob. (1987).

The involucre of the Belize species is eximbricate with mostly 2 series of equal, obovate bracts with broadly rounded tips (Fig. 2C). While *Fleischmannia* often has the superficial appearance of being eximbricate, an appearance allowed for in many keys (King and Robinson 1987), it is always actually subimbricate with the involucral bracts in more than two series.

The receptacle of the Belize species is highly conical as seen in two heads in which the receptacle shows without dissection (Fig. 1C), while that of *Fleischmannia* is plane to scarcely convex.

The corolla has a basal tube closely investing the style and an abruptly expanded campanulate limb, differing from consistently funnelform shape in *Fleischmannia*.

The carpopodium of the Belize plant has enlarged and elongate cells (Fig. 1F), a feature different from the smaller subquadrate cells in *Fleischmannia*.

There are a few more subtle distinctions from *Fleischmannia*:

The anther thecae are very short, and the apical anther appendages are slightly broader than long (Fig. 1E).

The cells of the corolla lobes are shorter and broader than those of *Fleischmannia* (Fig. 1D)

The pappus bristles are ca. ten in number and separated at the base (Fig. 2), a feature true of some *Fleischmannia*, but the bristles of the pappus are broad at the base unlike those in the *Fleischmannia* species which have five or ten non-contiguous bristles.

A few features are similar to those of *Fleischmannia*: the cells of the corolla lobes project at their distal ends on both surfaces of the lobes (Fig. 1D), the anther collars are very narrow and strongly transversely annulated, though the annulations do not completely obscure the crosswalls of the cells in the collars, the cells of the carpopodium have reasonably thick walls, and the style bases are neither enlarged nor papillose.

The most striking feature of the new genus is the high-conical and fistulose receptacle (Fig. 1C). How this structure functions is hard to determine, since there is no evidence of raised central florets in the flowering heads on the holotype. Careful examination of the photographs, however, shows a central cluster of corolla-like material and a possible exposed tip of receptacle in the center of the cluster (Fig. 1B). It seems possible that florets never fully develop on the distal part of the receptacle.

The position of the Belize species in the Eupatorieae is not resolved. Although a number of features are shared with *Fleischmannia*, and the two may have some phylogenetic relationship, the distinction of the species from anything in *Fleischmannia* is now certain. Furthermore, the new genus, cannot be placed in any of the other genera presently recognized in the tribe. It is particularly notable that few members of the Eupatorieae have a highly conical receptacle: *Isocarpha* R. Br. in which the receptacle is paleate, and *Praxelis* Cass, and *Eupatoriopsis* Hieron., both members of the subtribe Praxelinae, that have completely deciduous involucral bracts. The latter two also have 3-costate or obcompressed achenes.

In the general key to all the Eupatorieae genera in King and Robinson (1987) the new genus runs to couplet 137 on the basis of the articulated bases of the involucral bracts, the more than five florets in the capitula, the symmetrical corollas of the capitula, the well-developed apical anther appendage, the three to five-ribbed prismatic cypsela, the pappus of ten, subequal capillary, non-plumose, persistent bristles, the persistent involucre, the pedunculate capitula, the epaleate receptacles and the leaves subtending the peduncles not in pseudowhorls. Of the three genera in couplet 137, *Ageratina* Spach, *Gymnocondylus* R.M.King & H.Rob. & *Fleischmannia*, all differ from the new genus by their plane or slightly convex receptacles. The first two differ by their expanded style bases, and *Fleischmannia* differs as indicated above. Because of these characteristics and because of the generally distinctive aspect of the specimen, the species is placed here in another new genus of the Eupatorieae which we name *Zyzyura*.

## Taxonomic treatments

### Zyzyura

H. Rob. & Pruski gen nov.

urn:lsid:ipni.org:names:77124260-1

http://species-id.net/wiki/Zyzyura

Differs from *Fleischmannia* by the conical and fistulose receptacle, the eximbricate rather ‘Piquerian’ involucre, the slender-based corolla with broadly campanulate limb, and elongate cells of the carpopodium.

#### Type.

*Fleischmannia mayana* Pruski.

#### Description.

Decumbent epilithic herbs rooting at proximal nodes, prostrate portions 5–20 cm long, with short internodes, mostly glabrous, ascending portions 15–30 cm tall, with few small hairs, with long basal internodes; stems narrowly fistulose. Leaves (Fig. 1A) opposite, petioles mostly 3–5 mm; blades deltate in outline, mostly 4–7 mm long and wide, 3-7-lobed with sinuses 1/3-1/2 to midvein, basal margin truncate or subtruncate, triplinervate at base, margins of lobes often slightly notched, sparsely pilose adaxially, paler and hairless with crowded glandular dots abaxially. Inflorescence (Fig. 1B) terminal on ascending portions, ascending parts not branched below, distally loosely branching with 3–10 capitula, with few minute opposite to subopposite bracteoles; peduncles mostly 0.5–1.8 cm long. Capitula discoid, broadly hemispherical, to 7 mm high and wide; involucres (Fig. 1C) eximbricate; bracts c. 16 in mostly 2 series, subequal, obovate, with obtuse to rounded tips, chartaceous-becoming more scarious distally, bicostate proximally, few shorter outer bracts, scarcely spreading at maturity, essentially glabrous; receptacle (Fig. 1C) high-conical, fistulose, epaleate, without evidence of fully developed florets born on distal part. Florets 20–23; corollas white, 2.2–2.3 mm long, mostly glabrous, sparse glandular dots on lobes, immediate base dilated, thickly ribbed, narrowed to a slender tube closely investing the style, limb abruptly ampliate, campanulate, lobes (Fig. 1D) deltate, with intermarginal ribs, cells projecting at distal ends on inner and outer surface; anther collars slender, with numerous annular thickenings that do not completely obscure transverse cell walls; anther thecae (Fig. 1E) c. 0.5 mm long; apical appendage slightly broader than long. Style base without expanded node, papillae or hairs, style appendages thickened, densely papillose. Cypsela (Fig. 2) 1.2–1.3 mm long, somewhat fusiform, 3–5 costate, with few scabrae on ribs; carpopodium (Fig. 1F) broadened, with distinct projecting upper rim, cells enlarged and elongate with moderately thickened walls; pappus iniserate (Fig. 2) with c. 10 persistent capillary bristles, reaching to approximately the base of corolla lobes, broadened but not contiguous at base, scabridulous, narrowed to apex. Pollen c. 18 μm in diam. in fluid.

#### Distribution.

The genus contains only the single species that may be endemic to the type locality in the Maya Mountains of the Cockscomb range in Belize.

#### Ecology.

Cited as epilithic.

#### Etymology.

Contrived name (no meaning).

#### Specimens examined.

*Zyzyura mayana* (Pruski) H. Rob. & Pruski, comb. nov. (IPNI ID: urn:lsid:ipni.org:names:77124264-1), basionym: *Fleischmannia mayana* Pruski in Pruski and Clase, Phytoneuron 2012-32: 6. 2012. Presently known only from Victoria Peak in the Cockscomb range of the Maya Mountains in Belize (*Brewer & Pau 3349*, holotype MO).

## Supplementary Material

XML Treatment for Zyzyura
